# Associations between BMI and home, school and route environmental exposures estimated using GPS and GIS: do we see evidence of selective daily mobility bias in children?

**DOI:** 10.1186/1476-072X-14-8

**Published:** 2015-02-06

**Authors:** Thomas Burgoine, Andy P Jones, Rebecca J Namenek Brouwer, Sara E Benjamin Neelon

**Affiliations:** Department of Community and Family Medicine, Duke University Medical Center, 2200 W Main St, DUMC 104006, Durham, NC 27705 USA; Duke Global Health Institute, Duke University, 310 Trent Hall, Durham, NC 27710 USA; UKCRC Centre for Diet and Activity Research (CEDAR), MRC Epidemiology Unit, University of Cambridge School of Clinical Medicine, Box 285 Institute of Metabolic Science, Cambridge Biomedical Campus, Cambridge, CB2 0QQ UK; Norwich Medical School, University of East Anglia, Norwich, NR4 7TJ UK

**Keywords:** Environmental exposure, Activity space, Body mass index (BMI), Global positioning systems (GPS), Geographic information systems (GIS), Selective daily mobility bias

## Abstract

**Background:**

This study examined whether objective measures of food, physical activity and built environment exposures, in home and non-home settings, contribute to children’s body weight. Further, comparing GPS and GIS measures of environmental exposures along routes to and from school, we tested for evidence of selective daily mobility bias when using GPS data.

**Methods:**

This study is a cross-sectional analysis, using objective assessments of body weight in relation to multiple environmental exposures. Data presented are from a sample of 94 school-aged children, aged 5–11 years. Children’s heights and weights were measured by trained researchers, and used to calculate BMI z-scores. Participants wore a GPS device for one full week. Environmental exposures were estimated within home and school neighbourhoods, and along GIS (modelled) and GPS (actual) routes from home to school. We directly compared associations between BMI and GIS-modelled versus GPS-derived environmental exposures. The study was conducted in Mebane and Mount Airy, North Carolina, USA, in 2011.

**Results:**

In adjusted regression models, greater school walkability was associated with significantly lower mean BMI. Greater home walkability was associated with increased BMI, as was greater school access to green space. Adjusted associations between BMI and route exposure characteristics were null. The use of GPS-actual route exposures did not appear to confound associations between environmental exposures and BMI in this sample.

**Conclusions:**

This study found few associations between environmental exposures in home, school and commuting domains and body weight in children. However, walkability of the school neighbourhood may be important. Of the other significant associations observed, some were in unexpected directions. Importantly, we found no evidence of selective daily mobility bias in this sample, although our study design is in need of replication in a free-living adult sample.

**Electronic supplementary material:**

The online version of this article (doi:10.1186/1476-072X-14-8) contains supplementary material, which is available to authorized users.

## Background

The aetiology of obesity is complex and multifaceted, and likely the product of a number of factors at individual, social and environmental levels [1]. Children’s dietary and physical activity behaviours, and therefore their body weight, may be partly shaped by a range of food, physical activity and built environment exposures [2, 3], which offer the opportunity to consume as well as expend energy. Neighbourhood characteristics linked to health have been recently implicated in design theories such as ‘New Urbanism’, ‘Smart Growth’ and ‘Neotraditonalism’ [4]. These describe the importance of walkable, pedestrian-orientated neighbourhoods, with well-connected streets, mixed land uses and good access to local amenities such as stores selling healthy food and green space. Such environmental characteristics have been hypothesised to act either directly (for example through promotion of physical activity), or via a pathway involving increased sense of community and social cohesion on behavioural outcomes and health [5]. These urban design principles reinforce the role of the planner in public health promotion. However, we need to better understand the extent to which exposures of this kind contribute to adiposity in children.

Recent reviews have noted a number of significant associations between neighbourhood food and built environment characteristics and body weight in children [6–10]. For example, greater proximity to convenience stores has been associated with higher body mass index [11], and greater density of recreational facilities with lower odds of overweight [12]. However, unexpected associations between environmental exposures and bodyweight have also been reported [13, 14]. In a recent systematic review of children’s green space access and physical activity [15], only 6 of the 14 studies identified indicated positive associations. Overall, the evidence base remains inconclusive, perhaps because studies often fail to simultaneously capture multiple environmental exposures related to both energy intake (such as food outlet access) and energy expenditure (such as green space access) [3, 16].

Studies also tend to focus exclusively on environmental exposures within home ‘neighbourhoods’. While definitions of neighbourhood vary between studies [17], ranging from buffers around the home address, to an administrative boundary of residence, to measures accounting for perceived neighbourhood boundaries, the use of any such definition invokes the notion of the ‘residential trap’ [18]; the assumption being that only the ‘local’ matters for health [19]. We know however that children spend a substantial amount of time outside of their home neighbourhood, and are as such spatially polygamous, in their simultaneous experience of and interaction with multiple spatial contexts [20]. One study found that adolescent girls spent more than a third of their waking hours more than 1 km from their homes [21]. Another demonstrated that boys, and children living in rural areas more generally, tended to roam frequently beyond their home 800 m street network defined neighbourhoods and engaged in more vigorous physical activity in these non-home locations [22]. Others have also found substantial contributions to daily levels of moderate to vigorous physical activity (MVPA) while travelling outside of home neighbourhoods [23, 24].

The high degree of spatial polygamy demonstrated even among children, makes it clear that “the human scale of activity no longer appears to coincide with the local scale of the residential neighbourhood” [2, 20]. This recognition has led to the development of the concept of the ‘activity space’ [25]. Activity spaces contain “the subset of all locations within which an individual has direct contact as a result of his or her day-to-day activities” [26], as bound by time, obligational and transport constraints [27]. For social and behavioural scientists in particular, the polycentric environmental exposures associated with key daily anchor points and movements between these locations within activity spaces, are potentially critical [20, 28]. In particular, behaviours practised in these wider non-home contexts have the potential to confound exposure-outcome models based on the home neighbourhood only. This is because such behaviours are erroneously attributed to a home exposure, when in fact they were undertaken in a non-home setting, which may be radically different in terms of its environmental characteristics [29].

Schools for example, are often cited as a potentially important non-home setting within activity spaces [2]. Children spend a lot of time in these locations, around which unhealthy food outlets have also been shown to cluster [30, 31]. Again, unobserved environmental exposure to food and built environments in the school setting, may act as potentially important source of confounding in home only models of individual-environment associations. Moreover, while associations have been observed between school-based built and food outlet exposures, dietary outcomes [32–35], and odds of overweight and obesity [36], associations with body mass index (BMI) have not been comprehensively tested. In a recent UK study, Harrison *et al.*[37] suggested that associations between school-based unhealthy food outlet access, green space availability and mixed land use, with lower body weight may exist, but predominantly for girls.

Routes between home and school, and their associated environmental exposures are also increasingly considered as important activity space correlates of behaviours and adiposity [28, 38]. These routes may provide important opportunities to access food outlets and physical activity facilities, while habitual behaviours may develop as a result of repeated daily exposure to the same journey environments. However, consistent evidence of the association between journey exposures to food and built environment characteristics and body weight has again not been found [37, 39, 40]. This may be because, despite the importance of these studies in considering previously neglected exposure settings and therefore advancing toward a more complete assessment of cumulative daily environmental exposure, routes to school have mostly been modelled based on the shortest street network distance between home and school [37, 39, 40], with only one study accounting for transport mode choice [29].

Global positioning systems (GPS) devices have been used to record actual routes to school [23, 41]. GPS devices allow calculation of actual environmental exposure, and are therefore a potentially powerful tool for advancing activity space exposure assessment, beyond the use of modelled GIS routes. It has also been shown that modelled GIS and actual GPS routes are not necessarily equal, in terms of the routes themselves or their associated exposures [42, 43]. Route selection remains a multifaceted decision, based on a range of factors including time, habitual and personal commitments, perceptions of safety and mode of transport [44–46]. However, because routes are so highly modifiable, the use of actual GPS routes in exposure assessment may hold implications for causal inference, described as ‘selective daily mobility bias’ [27, 47]. For example, routes (and thus ‘exposures’) may be selected based on preferences related to BMI, such as the desire to access food. Resulting associations between BMI and food outlet exposure may therefore reflect participant preferences to be ‘exposed’, more so than the effects of the exposure *per se*. Using GPS, Harrison *et al.*[43] showed how food outlet exposure was greater on the way home from school than on the way to school in a sample of UK school children, a finding that may reflect some degree of this route self-selection. The suggestion is therefore that outcomes such as BMI might be better explained by actual GPS route exposures (which may reflect behavioural preferences and are readily modifiable) than by modelled GIS route exposures, however this hypothesis has not yet been tested in the literature.

This study aimed to assess home, school and journey exposures to food, physical activity and built environments, and their associations with measured BMI in a sample of school-aged children in North Carolina (NC). We used GPS devices to accurately capture journeys to school, and their associated exposures, in an evolution from previous modelling of these routes using GIS. As part of this, we directly address Chaix *et al.’*s [47] implication of selective daily mobility bias through using GPS data, through a formal comparison of GIS modelled versus GPS actual route environmental exposures. According to the selective daily mobility bias thesis, GPS derived actual route environmental exposures should better predict BMI than their modelled exposure equivalents.

## Methods

### Study participants

The study sample was drawn from children participating in an evaluation study of a natural experiment, using baseline data only. Mebane on the Move is a grassroots campaign, designed to promote healthier lifestyles and prevent obesity through physical activity in the small town of Mebane, NC. Mebane on the Move is comprised of a number of initiatives, from establishing walking trails, adding footpaths to roads, and adding pedestrian crossings to streets, to free fitness classes for town residents and the formation of running clubs in schools. This intervention has been described in detail elsewhere [48]. As part of an evaluation of this natural experiment, a demographically matched yet geographically distinct ‘comparison’ town (Mount Airy, NC) was identified. The researchers recruited a sample of children aged 5–11 years and their parents through three schools in each town (six schools in total).

A sub-sample of 94 children across Mebane and Mount Airy study sites agreed to wear a Qstarz BT-Q1000X GPS device on the left hip, over one full week (five weekdays and two weekend days) during baseline assessments in 2011. GPS devices recorded precise geographic locations at 60 second intervals (epochs). In this sub-sample, children’s heights and weights were also measured by trained research staff (using a Seca 124 portable stadiometer and a Tanita BWB-800 portable scale, respectively), and age-specific BMI z-scores calculated relative to growth charts from the US Centers for Disease Control and Prevention (CDC) [49]. Parents returned an accompanying self-report questionnaire, which included a range of socio-demographic questions, as well as home address and school attended by their child. Parents also reported their child’s weekly frequency of travel to school by different transport modes. We geocoded addresses in a GIS (ArcGIS 10, ESRI Inc., Redlands, CA), using an address locator derived from 2010 US TIGER street network data. For this study, we defined the school location according to the precise location of the main school entrance, determined using aerial imagery, as in previous work [37]. This study was conducted according to the guidelines laid down in the Declaration of Helsinki and all procedures involving participants were approved by the Duke University Medical Center Institutional Review Board. Parents of children provided written and informed consent and children provided assent.

### Environmental exposures

#### Defining environments

**Figure 1:**
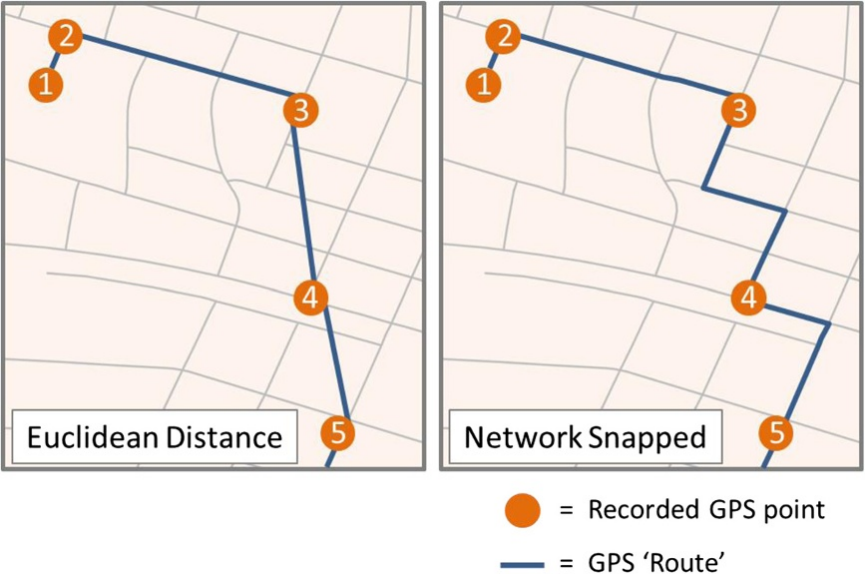
**Recorded GPS data points, joined via the shortest Euclidean distance (left), and constrained (‘snapped’) to the TIGER street network (right).** This illustration shows how snapping to the street network likely results in a more accurate estimation of the route travelled.

In order to quantify actual environmental exposure while travelling to and from school, we first exported GPS coordinates from the GPS devices, merged them into a single database, and subsequently stratified by school attended, participant ID and calendar date. The GPS points were then plotted using GIS software. Using school day start and end times as a guide, journeys to and from school were then isolated by removing GPS points before children left for school in the morning, after children arrived at school until the time at which they left school, and after children arrived back home. The GPS tracks were manually trimmed in this way for all journeys (n = 775), for all children (n = 94). In order to produce a route to and from school, we joined these points using TIGER street network data as a framework. As a result of GPS sampling at 60 second epochs, it was important to ‘snap’ GPS points along the road network in this way, as opposed to simply connecting points with straight lines, based solely on shortest Euclidean distance (Figure 1). Modelled GIS routes were defined using the shortest distance along the street network between homes and schools. We buffered GPS tracks and GIS routes by 100 m, within which to estimate environmental exposure. There is growing precedent for use of this 100 m route exposure delineation in the literature [37, 39, 50].

Complementing route exposure estimates, we also calculated potential environmental exposures in home and school neighbourhoods using GIS. We defined this potential exposure in two ways, depending on the environmental attribute we intended to capture (see Table 1). One approach defined ‘neighbourhoods’ as 800 m street network buffers around home and school locations, using the TIGER street network data. Precedent for the use of 800 m street network neighbourhoods has been set in the literature [31, 51]; for a child, 800 m represents about a 10 minute journey if travelling on foot [22]. However, many homes and schools were likely to have no or few facilities within these 800 m neighbourhoods, thereby necessitating the use of facilities beyond these limits. Our second approach was therefore to define potential access using inverse distance weighting (IDW). This technique does not require a neighbourhood extent to be formally defined, and as such can also help to overcome concerns regarding the importance of the very local [18, 19]. Using this approach, all discrete point facilities (food outlets and physical activity locations) within a 6 km street network cut-off contribute to exposure, with the inverse distance (1/*d*) between point facilities (*i*) and homes or schools (*j*) then weighted according to a suggested distance decay parameter (*k*) of 2 [52]. Precedent for the use of this inverse distance weighting approach has been set previously [37].

**Table 1:** **Details of exposure metrics, and descriptive statistics for home, school and route environments**

Variable	Description - home/school neighbourhoods	Description – routes	Data source	Descriptive statistics (Mean, SD)			
				Home	School	Modelled route	Actual route
***Access to facilities:***							
Takeaway food outlets	The inverse distance weighted sum of distance to all outlets/facilities within 6 km of home/school	Number of outlets/facilities along the route, divided by route length	A, B, C	3.29 (2.84)	4.40 (2.10)	0.33 (0.52)	0.19 (0.37)
All food outlets				6.38 (5.10)	8.44 (3.08)	0.64 (0.96)	0.37 (0.51)
Physical activity facilities				0.81 (0.62)	1.13 (0.34)	0.09 (0.16)	0.07 (0.10)
Green space^a^	Area of green space within neighbourhood as a percentage of neighbourhood area	Area of green space along the route, divided by route length	C, D	62.21 (15.15)	51.66 (11.48)	111.16 (25.50)	116.98 (191.84)
***Road safety:***							
Density of fatal road traffic accidents	Number of fatal road traffic accidents in the neighbourhood 2002–2011, divided by total length of roads within the neighbourhood	Number of fatal road traffic accidents along the route 2002–2011, per km of route length	C, E	0.03 (0.07)	0.04 (0.05)	0.14 (0.34)	0.14 (0.20)
Proportion of roads that are major roads	Length of major roads within the neighbourhood, divided by total length of roads within the neighbourhood	Percentage of the route that is along a major road	C	0.09 (0.13)	0.12 (0.09)	39.95 (33.53)	36.00 (24.57)
***Street connectivity:***							
Effective walkable area and route length ratio	Effective walkable area: ratio of the area within an 800 m street network distance of a location to the total area within an 800 m Euclidean radius	Route length ratio: ratio of length of route to the Euclidean distance between home and school	C	0.18 (0.07)	0.25 (0.02)	1.16 (0.25)	2.78 (2.12)
Connected node ratio	Ratio of junctions to junctions and cul-de-sacs	n/a	C	0.79 (0.11)	0.90 (0.06)	n/a	n/a
***Land use mix:***							
Herfindahl-Hirschmann Index^b^	The sum of squares of the percentage of each land use type in the neighbourhood	The sum of squares of the percentage of each land use type along the route	D	3716.44 (148.55)	3686.80 (730.00)	3564.09 (817.50)	3447.12 (703.70)

Data sources: A = ReferenceUSA 2011 [53]; B = Geo-coded by trained researcher, 2011; C = Topologically Integrated Geographic Encoding and Referencing (TIGER) 2011 [54]; D = US National Land Cover Database (NLCD) 2006 [55]; E = National Highway Traffic Safety Administration (NHTSA) Fatality Analysis Reporting System (FARS) 2002–2011 [56].

^a^US NLCD land uses summed to represent ‘green space’: developed open space, woodland (combining deciduous forest, evergreen forest, mixed forest) and grassland.

^b^Nine different US NLCD land uses included: open water, developed open space, developed low-high intensity, barren land, woodland, scrubland, grassland, farmland and wetland.

#### Characterising environments

Table 1 provides a description of the exposures calculated, calculation details, and details of data sources used. The relationships between these exposures, which are hypothesised to both encourage and discourage energy intake and expenditure in children, and BMI, forms the basis of the analysis in this work. Firstly, using location data provided by Reference USA, a commercial data company from whom we purchased data for this study, we calculated potential access to all food outlets and takeaway food (‘fast food’) outlets using IDW. We used North American Industry Classification System (NAICS) codes, also provided by Reference USA, to identify takeaway food outlets: we categorised ‘Limited-service restaurants’ and ‘Snack and non-alcoholic beverage bars’ (NAICS codes 722110 and 722213) as unhealthy sources of takeaway food. To confirm primary food outlet classification, where this was otherwise not clear, we made phone calls and field visits. We also calculated access to formal physical activity facilities, which included ‘community centres’ and ‘sports facilities’ (locations ground truthed by a trained researcher), in this way.

We estimated other attributes of the physical activity and built environments within our 800 m street network home and school neighbourhoods. We calculated neighbourhood percent green space [37, 57, 58]; density of fatal road traffic accidents as a measure of road safety [37]; proportion of ‘major’ roads using TIGER road type definitions (defining S1100 ‘primary road’ and S1200 ‘secondary road’ segments as ‘major’) [37, 59, 60]; neighbourhood effective walkable area [59, 60] and connected node ratio [61, 62], as measures of walkability; and land use mix, which is also a measure of walkability [62], using the Herfindahl-Hirschmann Index [37] and considering nine land use types.

With the exception of the connected node ratio, we also characterised route exposure to the environmental traits outlined above. Some modification of these home and school metrics was necessary (see Table 1). For example, ‘effective walkable area’ was calculated for homes and schools, but was adapted to the equivalent ‘route length ratio’ for routes. We averaged actual GPS exposures where more than one journey was recorded per child, resulting in an exposure estimate representative of that experienced throughout the time the child wore the GPS device.

### Statistical analysis

Descriptive statistics were calculated for the full sample and stratified by sex. We used the following strategy to estimate associations between potential environmental exposures at home and school, potential exposures along modelled GIS routes to school, and actual exposures along GPS routes, with continuous BMI z-score. As many of the environmental exposure metrics created were not normally distributed, we transformed all of our environmental variables into tertiles of exposure. The continuous extents of exposure tertiles are presented in Additional file 1: Table S1. Firstly, we present unadjusted associations, as mean BMI z-scores across tertiles of exposure, with a test for trend (Pearson’s correlation analysis). We tested for differences in mean BMI z-score between GIS-modelled and GPS-actual route methods, within route exposure tertiles, using analysis of variance (ANOVA). Secondly, we conducted bivariable linear regression analyses, regressing BMI z-score onto exposure tertiles (relative to the least exposed reference tertile 1), while adjusting for sex, and the highest educational attainment of the head of the household. To keep these models as parsimonious as possible, education was selected as a measure of socio-economic status at the expense of income, while race was not included as a covariate because there was little heterogeneity in race in this sample. Using age-specific BMI z-scores meant that we did not need to add age as a covariate in these models. We tested for trend in these relationships in parallel models with continuous exposures (data not presented), and tested for differences in means of predicted BMI z-scores between GIS-modelled and GPS-actual route exposure tertiles using ANOVA. All analyses were conducted using PASW Statistics 21 (PASW Statistics Inc., Chicago, 2009).

## Results

Descriptive statistics for environmental exposures at home, at school and while commuting to and from school are presented in Table 1. Exposure to all types of food outlets, takeaway food outlets, as well as formal physical activity facilities was greater at school than at home. Green space access was greater at home, while in general school neighbourhoods were more walkable (better connected, with fewer cul-de-sacs in particular). Environmental exposures along actual and modelled routes were similar, but the greater route length ratio for actual GPS routes (2.78) compared to modelled GIS routes (1.16) suggests that the former were longer on average.

The characteristics of the study participants are shown in Table 2. There were a slightly greater number of boys in the sample, who also had higher median BMI z-scores (boys, 0.95; girls, 0.80). The median age for the sample was 8 years. One third (n = 31, 33.0%) of participants belonged to households with annual incomes over $90,001, while the majority of parents held at least a college degree (n = 66, 70.2%). Each week, the majority of study participants (n = 91, 96.8%) travelled inactively to school by either car or bus, or included at least some sort of motorised transport in their usual weekly commute patterns.

**Table 2:** **Descriptive statistics for NC on the Move analytic sample study participants (n = 94)**

		Count (%), or median, IQR, unless otherwise stated		
		Girls	Boys	All
**Number of children**		46 (48.9)	48 (51.1)	94 (100.0)
**Number of trips**		392 (50.6)	383 (49.4)	775 (100.0)
**Age in years (mean, SD)**		7.96, 1.62	8.13, 2.10	8.04, 1.85
**Child BMI z-score** ^**a**^		0.80, −0.05–1.78	0.95, 0.14–1.67	0.88, −0.04–1.72
**Child race**	White	38 (82.6)	41 (85.4)	79 (84.0)
**Household income**	Up to $15,000	2 (4.3)	5 (10.4)	7 (7.4)
	$15,001 to $30,000	5 (10.9)	10 (20.8)	15 (16.0)
	$30,001 to $60,000	10 (21.7)	4 (8.3)	14 (14.9)
	$60,001, to $90,000	10 (21.7)	16 (33.3)	26 (27.7)
	More than $90,001	19 (41.3)	12 (25.0)	31 (33.0)
**Parent education level**	1st to 8th grade	1 (2.2)	0 (0.0)	1 (1.1)
	9th to 12th grade	2 (4.3)	3 (6.3)	5 (5.3)
	Vocational or some college	8 (17.4)	14 (29.2)	22 (23.4)
	College graduate	19 (41.3)	13 (27.1)	32 (34.0)
	Graduate or professional school	16 (34.8)	18 (37.5)	34 (36.2)
**Child’s most frequent mode of travel to school per week**	On foot	1 (2.2)	2 (4.2)	3 (3.2)
	Bus	12 (26.1)	14 (29.2)	26 (27.7)
	Car	25 (54.3)	27 (56.3)	52 (55.3)
	Multi-modal^b^	8 (17.4)	5 (10.4)	13 (13.9)

^a^BMI z-scores calculated relative to age-specific US national height and weight distributions, from the Centers for Disease Control and Prevention (CDC).

^b^Defined as the equal use of two or more different travel modes for journeys to and from school per week. NB All multi-modal commute patterns contained at least one form of motorised transport in this sample.

Table 3 shows unadjusted associations for BMI z-score across tertiles of home, school and commuting environmental exposures. Increased exposure to all and particularly takeaway food outlets was associated with increased mean BMI, but only around the home. Greater home access to physical activity facilities and greater school access to green space was associated with significantly higher BMI. Greater school walkability (effective walkable area) was associated with lower mean BMI, but associated with higher mean BMI in home neighbourhoods. Similarly, greater school land use mix (higher walkability) was associated with significantly lower mean BMI at school, but higher BMI at home. In terms of route exposures, whether based on actual GPS routes or modelled GIS routes, all associations with BMI were null. There were no significant differences in mean BMI between GPS-actual and GIS-modelled approaches to estimating environmental exposures.

**Table 3:** **Unadjusted mean BMI z-scores within tertile of environmental exposure, with tests for trend**

		Mean BMI z-score per exposure tertile (Pearson’s correlation co-efficient)			
	Tertile	Home	School	Modelled journey	Actual journey
**All food outlets**	1 (least exposed)	0.606	1.031	0.852	0.811
	2	0.710	0.592	0.700	0.979
	3	1.157 (0.208)**	1.018 (−0.081)	0.863 (−0.053)	0.671 (−0.104)
**Takeaway food outlets**	1 (least exposed)	0.722	1.031	0.924	0.737
	2	0.590	0.592	0.613	0.877
	3	1.161 (0.207)**	1.018 (−0.091)	0.790 (−0.060)	0.851 (−0.142)
**Physical activity facilities**	1 (least exposed)	0.593	1.179	0.721	0.551
	2	0.903	0.324	1.061 (0.052)	0.727
	3	0.967 (0.177)*	0.681 (−0.140)	-	1.197 (0.104)
**Green space**	1 (least exposed)	0.982	0.506	1.012	0.930
	2	0.651	0.681	0.761	0.859
	3	0.828 (−0.068)	1.379 (0.291)***	0.690 (−0.024)	0.676 (−0.131)
**Density of fatal road traffic accidents**	1 (least exposed)	0.836	0.926	0.795	0.570
	2	0.763 (−0.084)	0.681 (−0.094)	0.885 (−0.122)	0.857
	3	-	-	-	1.038 (−0.020)
**Proportion of roads that are major roads**	1 (most walkable)	0.655	0.640	0.765	0.862
	2	1.306	0.976	0.617	0.557
	3	0.952 (0.094)	0.747 (0.095)	1.085 (0.126)	1.056 (0.062)
**Effective walkable area/Route length ratio (for journeys)**	1 (least walkable)	0.548	1.379	1.066	0.773
	2	0.808	0.681	0.663	0.850
	3	1.110 (0.180)*	0.506 (−0.289)***	0.743 (−0.083)	0.842 (0.071)
**Connected node ratio**	1 (least connected)	0.655	1.031	-	-
	2	0.905	0.681	-	-
	3	0.912 (0.027)	0.843 (−0.059)	-	-
**Herfindahl-Hirschmann Index**	1 (least mixed)	0.559	1.031	0.554	0.506
	2	0.886	0.976	1.055	1.064
	3	1.020 (0.175)*	0.413 (−0.171)*	0.849 (0.113)	0.888 (0.113)

***p < 0.01, **p < 0.05, *p < 0.1, with reference to test for trend (Pearson’s correlation analysis) between environmental exposure and BMI z-score.

Table 4 shows adjusted bivariable regression models for the relationships between home, school and journey environmental exposures and BMI. Greater school green space access was associated with significantly higher mean BMI (test for trend, β = 0.031, 95% CI 0.010, 0.051). Greater home walkability (effective walkable area) was associated with significantly higher mean BMI (β = 3.634, 95% CI 0.211, 7.056), whereas greater school walkability was associated with significantly lower BMI (β = −16.572, 95% CI −28.239, −4.904). All other adjusted associations were null. There were no significant differences in predicted BMI z-scores between GPS-actual and GIS-modelled approaches to estimating environmental exposures.

**Table 4:** **Predicted BMI z-score per tertile of environmental exposure, in home, school and route settings**

	β co-efficients for BMI z-score ^a^								
	Tertile	Home	95% CI	School	95% CI	Modelled journey	95% CI	Actual journey	95% CI
**All food outlets**	1 (least exposed)	REF	n/a	REF	n/a	REF	n/a	REF	n/a
	2	0.010	−0.577, 0.596	−0.246	−0.874, 0.382	−0.246	−0.885, 0.393	0.157	−0.435, 0.748
	3	0.425	−0.163, 1.013	0.136	−0.529, 0.802	−0.041	−0.611, 0.529	−0.154	−0.756, 0.449
**Takeaway food outlets**	1 (least exposed)	REF	n/a	REF	n/a	REF	n/a	REF	n/a
	2	−0.154	−0.743, 0.435	−0.246	−0.874, 0.382	−0.455	−1.143, 0.233	0.324	−0.288, 0.937
	3	0.347	−0.244, 0.939	0.136	−0.529, 0.802	−0.093	−0.630, 0.445	0.153	−0.441, 0.746
**Physical activity facilities**	1 (least exposed)	REF	n/a	REF	n/a	REF	n/a	REF	n/a
	2	0.128	−0.477, 0.732	−0.763	−1.484, −0.042	0.325	−0.200, 0.851	0.341	−0.232, 0.913
	3	0.359	−0.233, 0.950	−0.398	−0.938, 0.143	−	−	0.906	0.317, 1.494
**Green space**	1 (least exposed)	REF	n/a	REF	n/a	REF	n/a	REF	n/a
	2	−0.315	−0.897, 0.268	0.267	−0.283, 0.816	−0.253	−0.854, 0.347	−0.040	−0.627, 0.546
	3	−0.183	−0.790, 0.424	0.892**	0.269, 1.515	−0.296	−0.876, 0.284	−0.320	−0.913, 0.274
**Density of fatal road traffic accidents**	1 (least exposed)	REF	n/a	REF	n/a	REF	n/a	REF	n/a
	2	−0.109	−0.719, 0.501	−0.137	−0.627, 0.353	0.033	−0.494, 0.561	0.323	−0.262, 0.908
	3	−	−	−	−	−	−	0.366	−0.238, 0.970
**Proportion of roads that are major roads**	1 (most walkable)	REF	n/a	REF	n/a	REF	n/a	REF	n/a
	2	1.002	0.192, 1.812	0.507	−0.099, 1.114	−0.116	−0.708, 0.476	−0.305	−0.902, 0.292
	3	0.062	−0.474, 0.598	0.231	−0.434, 0.897	0.187	−0.426, 0.800	0.133	−0.462, 0.728
**Effective walkable area/route length ratio (for journeys)**	1 (least walkable)	REF	n/a	REF	n/a	REF	n/a	REF	n/a
	2	0.105	−0.500, 0.710	−0.625	−1.207, −0.044	−0.423	−1.019, 0.172	0.126	−0.474, 0.726
	3	0.610**	0.021, 1.200	−0.892**	−1.515, −0.269	−0.447	−1.053, 0.160	−0.026	−0.640, 0.588
**Connected node ratio**	1 (least connected)	REF	n/a	REF	n/a	−	−	−	−
	2	0.232	−0.364, 0.828	−0.140	−0.765, 0.485	−	−	−	−
	3	0.302	−0.285, 0.889	−0.005	−0.657, 0.647	−	−	−	−
**Herfindahl−Hirschmann Index**	1 (least mixed)	REF	n/a	REF	n/a	REF	n/a	REF	n/a
	2	0.360	−0.244, 0.963	0.139	−0.460, 0.738	0.597	−0.007, 1.201	0.578	0.000, 1.156
	3	0.493	−0.101, 1.086	−0.441	−1.102, 0.221	0.362	−0.230, 0.955	0.307	−0.276, 0.889

**p < 0.05, using tests for trend based on modelling continuous environmental exposures.

^a^β co-efficients represent BMI z-scores across tertiles of environmental exposure, relative to the least exposed tertile 1. All models control for parental education level and sex of child.

## Discussion

We explored associations between home, school and route exposures to food, built and physical activity environments, in relation to BMI, in a sample of 94 school-aged children in North Carolina, USA. We also addressed the potential impact of selective daily mobility bias through a formal comparison of modelled GIS versus actual GPS route environmental exposures. We found limited evidence of significant associations between BMI z-score and environmental exposures across home and school neighbourhoods, although greater school neighbourhood walkability was associated with significantly lower mean BMI. We observed no significant associations between BMI and modelled GIS nor actual GPS route environment exposures in this sample. In unadjusted and adjusted analyses, BMI z-scores were similar between exposure tertiles, whether based on modelled GIS or actual GPS routes between homes and schools.

This study addresses an important gap in the literature through being the first to formally compare associations between BMI and GIS modelled versus GPS actual environmental exposures. While GPS devices are increasingly considered a powerful tool for advancing exposure assessment beyond the use of modelled GIS routes, it was important to consider how GPS derived exposure might be vulnerable to ‘selective daily mobility bias’ [27, 47]. Should route choice have been heavily based on preferences related to BMI in this sample, we would have expected actual route exposures to have been more strongly associated with BMI than their modelled exposure equivalents. While associations with modelled GIS route exposures and BMI have been described elsewhere in children [37], we were unable to replicate such findings in this study using modelled GIS nor actual GPS route exposures. Therefore, there was no evidence of confounding whereby children with higher BMIs chose to travel to school via more ‘obesogenic’ routes, and vice versa for children with lower BMIs. In part, lack of evidence for both route exposure effects and selective daily mobility bias could be a reflection of the age of participants in this sample, as well as the types of journeys made, which may have reduced the likelihood of route self-selection. For example, when being driven to school (55% of participants travelled to/from school exclusively by car), route selection will be strongly influenced by the priorities of parents for food, physical activity or otherwise. There would have been an even greater lack of autonomy over route choice when using the school bus, which 27% of this sample used exclusively. While this study represents a first step in examining the potential for selective daily mobility bias when using GPS data, our work is in need of replication in a free-living sample of adults who are potentially more able to interact with their environments.

We did observe a significant adjusted association between land use mix in the school neighbourhood and BMI, suggesting that greater walkability in this setting was related to lower body weight. Methodologically, this finding reiterates the importance of accounting for non-home activity space environmental exposures in furthering our understanding of behaviours and health [20]. Similar associations, using similar measures of land use mix, have been observed elsewhere. For example, Harrison *et al.*[37] showed how greater land use mix was associated with lower body weight in children, in school neighbourhoods in the UK. These complementary findings support New Urbanism design approaches, and help us understand the potentially important role of the planner in promoting health through design [63]. Conversely, we observed significant positive associations between BMI and land use mix in home neighbourhoods, suggesting that these areas are less supportive of maintaining a healthy body weight. However, as the relationship between increased neighbourhood walkability and lower body weight is thought to be mediated through increased physical activity levels, this unexpected association might be explained by previous research demonstrating the large proportion of physical activity undertaken by children outside of the home neighbourhood [22–24]. While greater school green space exposure was associated with having significantly higher mean BMI, this could be explained in part by exposure misclassification. For example, our green space measure is likely to have included areas that were privately owned and were thus inaccessible, as well as areas that were physically inaccessible or may have been perceived as unsafe and therefore ‘off limits’ to children [64]. Green spaces may also encourage sedentary rather than moderate or vigorous physical activity, which would be more likely to be translated into higher body weight [65].

In unadjusted analyses, we did find that home access to takeaway food outlets was associated with higher BMI. This observed association, although null when adjusting for parental education and sex, is consistent with previous research findings [11, 37, 66], and suggests a pathway linking takeaway food outlet access, via unhealthy dietary behaviour to increased body weight. Recent data from London, UK, showed that 30% of fried chicken shop customers were less than 12 years old, demonstrating patronage of takeaway food outlets even in this young age group [67]. This unadjusted association was observed for the home environment only, which could be due to a number of factors, including restrictions on leaving school grounds at lunch to access takeaway food outlets, and use of modes of transport between home and school that might have limited engagement with the school food environment before and after school.

This study has a number of limitations. For capturing actual route exposures, we acknowledge that the GPS tracks recorded may not be representative of those usually travelled. Indeed, participants may have altered their behaviours as a result of being included in the study, or, may have been traversing different routes to school during the study period simply by chance. While capturing GPS data over the course of a week adheres to precedents set within the literature, data collection over a longer time frame might help to ensure that routes captured are indicative of usual travel behaviour, although this needs further investigation. We also constrained the path between recorded GPS points to the street network, which may have introduced some random error into our estimations of route exposure. Our analysis may have been impacted by conventional limitations widely associated with the use of GPS devices and data, such as locational imprecision due to poor satellite coverage in built-up or wooded areas. Many modern smartphones are able to boost GPS trilateration accuracy in urban areas by interfacing with nearby Wi-Fi networks and cell towers. Such technology may be harnessed by researchers in the future, allowing a GPS user’s location to be more accurately determined than is currently possible.

For children living close to school, there was potential for overlap and therefore collinearity between home and school neighbourhood exposures. Overall however, the average correlation between home and school exposure estimates was weak (r_p_ = 0.033). We also acknowledge that while home, school, and commuting exposures are theoretically important determinants of behaviour, exposures during other times of day, for example after getting home from school, and on weekends, also represent theoretically important behavioural correlates and therefore potentially unobserved confounders of the associations with weight status tested here.

In our analysis we were unable to use GPS data to capture actual environmental exposures close to homes and schools, in the same way that we did for routes. However, in exploratory work we found that very little time was actually spent in the vicinity of outlets in these neighbourhoods, yielding insufficient data from which to model associations with BMI in comparison with potential home and school exposures. For example, the GPS data suggested that only nine children spent any time within 50 metres of any type of food outlet in their school neighbourhood, and just seven children spent any time around any food outlet at home. Time spent near physical activity facilities was equally limited.

We estimated visible environmental exposures along journeys to and from school by buffering routes by 100 m, according to established precedent in the literature [37, 39, 50]. However, we acknowledge that this definition of route exposure remains arbitrary and that our results may be sensitive to this selection. We averaged route exposures for children taking multiple routes to school, however some children wore their GPS devices, and therefore recorded more GPS tracks on more occasions than others. It is, however, unclear if or how disparities in wear time might bias our results.

While we identified some significant associations throughout, our relatively small sample size (n = 94) may not have allowed us to detect all meaningful associations present within the data, and so our results should be seen as exploratory. Where other studies have found sex-specific neighbourhood environment effects in children [8, 66], our limited sample size prevented us from stratifying our analyses. The need to transform our exposure variables into tertiles (due to them not being normally distributed) may have reduced the sensitivity of our analyses, and therefore further reduced our ability to detect significant associations. Especially given the age of participants in this sample, physical activity or dietary behaviours may be more closely related to these environmental exposures than BMI, however such outcome data were not available. Further limitations related to our sample of households include their relative affluence and mostly high levels of education, which may limit generalisability, as well as the high proportion of participants commuting to and from school by motorised transport, which may explain null associations between route exposures and body weight. These issues related to study design and sampling should be resolved in future work.

Our data on food outlet and formal physical activity locations was purchased from a commercial database (Reference USA), and despite extensive precedent for the use of such data in the literature, and the necessity of using such an ‘extensive secondary’ food environment data source [68], studies have suggested that datasets of this type may not represent a complete record of all outlets and facilities [69, 70]. Our food outlet classifications were based on outlet name and extensive internet research, guided by NAICS codes supplied by Reference USA along with the dataset. However we were not able to include a within-store audit of food types and products sold, and there is evidence that the accuracy of NAICS codes varies by store type [71]. It is likely that some of the food outlets we deemed unhealthy ‘takeaway’ food outlets also sold some healthier menu options, which may help to explain our null associations. While we verified food outlet type where necessary by phoning businesses, and in some cases conducting store visits, we were still not able to account fully for such within-store heterogeneity in this study.

## Conclusions

This study examined multiple estimates of environmental exposure throughout children’s activity spaces. We found few associations with measured BMI. Of those observed, some were in unexpected directions, such as the positive relationship between home neighbourhood walkability and body weight. However we did find a negative association between body weight and school neighbourhood walkability, which closely matches previous research findings. Importantly, we also found no evidence of selective daily mobility bias, as suggested by Chaix *et al.*[47], when utilising actual GPS route exposures as compared to modelled GIS route exposures. The use of GPS technology did not therefore appear to confound the associations between environmental exposures and BMI in this sample, although our assessment of this bias is now in need of replication in other studies with free-living adult samples, and in which exposure in wider activity spaces can be considered.

### Ethical approval

Participants gave written and informed consent and the study was approved by the Duke University Medical Center Institutional Review Board. All other data analysed was in the public domain.

## Electronic supplementary material

Additional file 1: Table S1: Continuous descriptive statistics for exposure tertiles. (DOCX 46 KB)
