# Supplementary material for: Associations between BMI and home, school and route environmental exposures estimated using GPS and GIS: do we see evidence of selective daily mobility bias in children?
Source: Int J Health Geogr. 2015 Feb 6;14:8. doi: 10.1186/1476-072X-14-8 (PMC4429367; doi:10.1186/1476-072X-14-8)
Supplement: Supplementary file 1 — Additional file 1: Table S1: Continuous descriptive statistics for exposure tertiles. (DOCX 46 KB) [file 12942_2014_636_MOESM1_ESM.docx]

|  |  | **Exposure range (continuous minimum-maximum)** | | | |
| --- | --- | --- | --- | --- | --- |
|  | **Tertile** | **Home** | **Work** | **Modelled Journey** | **Actual Journey** |
| **All food outlets**  Home/school: inverse distance weighted sum of distance to all outlets within 6km \| route: number of outlets along route divided by route length | 1 | 0-4.23 | 5.60-6.04 | 0-0 | 0-0.15 |
|  | 2 | 4.66-8.08 | 6.97-8.48 | 0.22-0.54 | 0.15-0.37 |
|  | 3 | 8.12-28.79 | 9.58-15.74 | 0.54-4.30 | 0.41-3.48 |
| **Takeaway food outlets**  Home/school: inverse distance weighted sum of distance to takeaway outlets within 6km \| route: number of takeaway outlet along route divided by route length | 1 | 0-1.65 | 2.83-3.05 | 0-0 | 0-0.02 |
|  | 2 | 1.93-4.08 | 3.29-3.85 | 0.13-0.36 | 0.03-0.17 |
|  | 3 | 4.10-15.67 | 5.43-9.43 | 0.38-2.51 | 0.17-2.78 |
| **Physical activity facilities**  Home/school: inverse distance weighted sum of distance to PA facilities within 6km \| route: number of PA facilities along route divided by route length | 1 | 0-0.29 | 0.55-1.05 | 0-0 | 0-0 |
|  | 2 | 0.37-1.20 | 1.19-1.19 | 0.09-0.58 | 0-0.7 |
|  | 3 | 1.24-2.66 | 1.28-1.71 | - | 0.08-0.44 |
| **Green space**  Home/school: area of green space as a percentage of neighbourhood area \| route: area of green space along route divided by route length | 1 | 7.69-57.49 | 38.21-40.54 | 46.23-103.05 | 42.37-87.19 |
|  | 2 | 57.66-66.14 | 41.55-53.25 | 103.20-122.79 | 87.23-105.97 |
|  | 3 | 66.67-95.03 | 65.13-69.84 | 122.93-241.16 | 106.58-1942.86 |
| **Density of fatal traffic accidents**  Home/school: number of fatal traffic accidents divided by total length of roads \| route: number of fatal traffic accidents along route divided by route length | 1 | 0-0 | 0-0 | 0-0 | 0-0.03 |
|  | 2 | 0.05-0.35 | 0.09-0.11 | 0.08-2.16 | 0.03-0.16 |
|  | 3 | - | - | - | 0.16-1.16 |
| **Proportion of roads that are major roads**  Home/school: length of major roads divided by total length of roads \| route: percentage of route that is a major road | 1 | 0-0 | 0-0.07 | 0-17.72 | 0-24.29 |
|  | 2 | 0.07-0.13 | 0.07-0.13 | 19.41-55.61 | 25.26-46.12 |
|  | 3 | 0.13-0.44 | 0.19-0.29 | 55.74-104.94 | 47.97-96.73 |
| **Effective walkable area/Route length ratio (for journeys)**  Home/school: ratio of the area within an 800m street network distance to the area within an 800m Euclidean radius \| route: ratio of length of route to Euclidean distance | 1 | 0.06-0.15 | 0.21-0.23 | 0.81-1.06 | 0.08-1.53 |
|  | 2 | 0.15-0.20 | 0.24-0.25 | 1.06-1.14 | 1.57-2.54 |
|  | 3 | 0.20-0.39 | 0.26-0.28 | 1.16-2.11 | 2.60-12.60 |
| **Connected node ratio**  Home/school: ratio of junctions to junctions and cul-de-sacs \| route: not applicable | 1 | 0.56-0.74 | 0.81-0.81 | - | - |
|  | 2 | 0.75-0.84 | 0.89-0.92 | - | - |
|  | 3 | 0.84-1.00 | 0.96-0.97 | - | - |
| **Herfindahl-Hirschmann Index**  Home/school/route: sum of squares of the percentage of each land use type | 1 | 2078.12-3045.92 | 2574.02-2926.12 | 2291.59-2960.73 | 2323.96-3000.63 |
|  | 2 | 3050.52-4255.31 | 3494.44-3831.05 | 2966.62-3892.39 | 3050.28-3804.54 |
|  | 3 | 4268.74-8585.27 | 4312.35-4983.26 | 3892.96-6852.68 | 3822.86-5358.39 |

**Additional file 1: Table S1:** Continuous descriptive statistics for exposure tertiles.
